# Dangerous Vinegar

**Published:** 1877-10

**Authors:** 


### Dangerous Vinegar.

The Board of Health of the District of Columbia has condemned five car-loads of vinegar sent there from Chicago, on the ground that it is not a genuine article, and is injurious to health. An analysis of the so-called vinegar has been made. It appears, according to the report of the Board of Health, that the vinegar contains $54\frac{54}{100}$ grains per gallon of anhydrous sulphuric acid, combined with lime, to form a sulphate of lime equivalent to $117\frac{26}{100}$ grains of gypsum per gallon, and besides that, five grains of free sulphuric acid per gallon. The Board also reports that this sample was taken from an invoice of more than 1,000 barrels brought there to be sold as vinegar, and that it is likely to find a ready sale on account of its low price. The report concludes as follows: "When we think that oil of vitriol (sulphuric acid) can be bought for five cents per pound, and that a pound of said acid would render a barrel of fluid as acid as the strongest vinegar, the wonder will cease that it is sold cheap. This, therefore, is a fraud upon commerce, and a dangerous substitute for vinegar." The fraud and danger are more general than the great mass of people will readily believe. It is asserted that probably one-half the vinegar sold at city groceries is a rank poison, with either sulphuric or other objectional acids for its base.—*Med. and Surgical Reporter.*

